# Clinical Study of Acupotomy for Knee Osteoarthritis Based on the Meridian-Sinew Theory: A Randomized Controlled Clinical Trial

**DOI:** 10.1155/2021/3987002

**Published:** 2021-11-18

**Authors:** Zhijuan Hua, Han Deng, Huang Tang, Zhizhong Ruan, Pei Wang, Min Zhang, Hui Ma, Ping Wang, Can Dong, Zhilan Huang, Hanqing Hong, Quan Zhou, He Zhou, Changcheng Cheng, Wanqi Lin, Cairong Zhang, Dechun Chen

**Affiliations:** ^1^Daishan Community Health Service Center, Nanjing, Jiangsu 210042, China; ^2^Nanjing University of Traditional Chinese Medicine, Nanjing, Jiangsu 210029, China; ^3^Nanjing Hospital of Chinese Medicine, Affiliated to Nanjing University of Chinese Medicine, Nanjing, Jiangsu 210029, China; ^4^Department of Acupuncture and Moxibustion, Nanjing Hospital of Traditional Chinese Medicine, Affiliated to Nanjing University of Chinese Medicine, Nanjing, Jiangsu 210001, China; ^5^Taihu Sanatorium of Jiangsu Province, Wuxi, Jiangsu 214100, China; ^6^School of Public Health, Guangdong Pharmaceutical University, Guangzhou, Guangdong, China; ^7^Department of Acupuncture and Moxibustion, The Second Affiliated Hospital of Nanjing University of Chinese Medicine, Nanjing, Jiangsu 210017, China; ^8^Nanjing First Hospital, Affiliated to Nanjing Medical University, Nanjing, Jiangsu 210006, China; ^9^Nanjing Integrated Traditional Chinese and Western Medicine Hospital, Nanjing, Jiangsu 210014, China

## Abstract

This study was performed to compare the effectiveness of acupotomy based on the meridian-sinew theory with acupotomy based on the anatomical theory in the treatment of knee osteoarthritis (KOA). A total of 124 patients with knee osteoarthritis were randomized into the meridian-sinew (MS) group (63 patients) and anatomy group (61 patients). In the MS group, acupotomy based on the meridian-sinew theory was performed. In the anatomy group, acupotomy based on anatomy was applied. Patients were subgrouped by TCM Constitutions. The Western Ontario and McMaster Universities (WOMAC) Osteoarthritis Index and visual analog scale (VAS) were used to evaluate treatment effectiveness. The results showed that VAS (*F* = 22.61, *p* < 0.01) and WOMAC (*F* = 24.84, *p* < 0.01) scores declined with time, and there was no significant difference between the two groups nor subgroups (Yang deficiency subgroup, Yin-Yang harmony subgroup, and the subgroup of the others). A total of 5 patients reported 6 cases of the minor adverse effect, and all patients achieved complete recovery without medical intervention. This study indicates that the effectiveness and safety of acupotomy based on the meridian-sinew theory are equivalent to that of acupotomy based on anatomy in KOA treatment.

## 1. Introduction

Knee osteoarthritis (KOA) is a chronic, progressive, and degenerative disease of the bone and joint characterized by cartilage degeneration, osteosclerosis, and hyperosteogeny. KOA is usually caused by aging, trauma, congenital joint abnormality, joint deformity, biomechanical factors, and endocrine metabolism [1]. KOA commonly occurs in people over 40 years old and can occur on both knees [2]. Epidemiological survey showed that KOA hared a high incidence in Chinese population aged over 60 (23.97%), and the incidence surged to 31.27% in people over 70 years old [3]. The prevalence of KOA was significantly higher in postmenopausal women [4, 5]. At present, the treatments of KOA mainly include pharmacologic options (such as nonsteroidal anti-inflammatory drugs [6], glucosamine sulphate and local analgesics, herbal medicines [7]), nonpharmacologic options, surgery, and complementary and/or alternative therapies. Treatments aim to relieve symptoms and restore the function of knee joint [1, 8]. One recent study shows that taking Tregocel® (containing curcuminoid and extracts of the herbs *Harpagophytum procumbens*, *Boswellia serrata*, *Apium graveolens*, and *Zingiber officinale*) is helpful for mild knee osteoarthritis [9]. However, such medicines could cause undesirable drug-related adverse reaction in the skin, upper gastrointestinal tract, brain, and kidney and increase risk of cardiovascular disease, while intraarticular injection and surgery may lead to infection and trauma [10, 11].

Acupotomy is a modern type of acupuncture that uses a needle knife combined with a flat scalpel at its tip and has been widely used in the treatment of KOA in China, especially in primary hospitals. The previous research studies and systematic evaluations also confirmed its effectiveness as an external treatment for KOA [12, 13]. In acupotomy therapy based on anatomy, a physician may use a “meridian-sinew scope” to observe local tissue and simultaneously use a needle knife to loosen and release adhesions [14]. It can relieve the pain, slow down the degeneration of articular cartilage, and maintain and improve joint function [14]. However, the meridian-sinew scope requires corresponding devices and accompanies with superior cost-burden. Whereas, acupotomy based on the meridian-sinew theory would shake off those limitations.

Meridian-sinew is one of the meridian subsystems. The word “meridian sinew” was translated from Jingjin, which also translated as “the 12 meridian sinews,” “muscle channels,” and “sinew channels.” In modern anatomy, meridian sinews are an accessory part of meridians and collaterals and is a system of deeply nourishing sinews, muscles, and joints. It can be viewed on the meaning of meridian sinews that the meridian-sinew system maintains the dynamic and static balance of joint motion. Physiologically, meridian sinews have the functions of connection, restraint, maintenance, and so on. Pathologically, the pathological changes of meridian sinews include sinew tension, sinew overrestriction, sinew cramp, sinew pain, and so on. The objective of this study is to provide a practical acupotomy method based on the meridian-sinew theory and exam the effectiveness and safety of this method in KOA treatment compared with that of acupotomy based on anatomy.

The health status of a person is strongly related to his or her constitution, which is a stable form of body structure and function as well as psychological state formed by the combination of innate endowment and acquired environment [15, 16]. It has been suggested that TCM constitution can reveal the sensitivity of patients to diseases, infer the regression of diseases, and use it to develop individualized treatment plans for patients [17].

In the previous study, the research group found that there were significant differences in the curative effects of patients. Most patients had reduced pain immediately after needle knife treatment, but some patients had increased pain after needle knife treatment and needed lidocaine injection to improve [18]. To seek the difference, this study added Identification Scale of TCM Constitutions to find the reason. Therefore, observing the physical distribution of KOA patients is worthwhile to explore its pathogenesis and provide reference for clinical syndrome differentiation and treatment of the disease.

## 2. Materials and Methods

### 2.1. Subjects

From January 2018 to December 2018, 124 patients with KOA in acupuncture departments of Nanjing Hospital of Chinese Medicine, Daishan Community Health Service Center, the Second Affiliated Hospital of Nanjing University of Chinese Medicine, and the Traditional Chinese Medicine Department of Nanjing First Hospital were recruited. All patients signed informed consent forms. We applied the following criteria for inclusion: (i) KOA diagnosed according to the diagnostic criteria of American College of Rheumatology [19, 20]; (ii) moderate or less pain (VAS < 7) during most days through the past month; (iii) agreed not to take any anodyne during treatment except analgesic or nonsteroidal anti-inflammatory drugs but not hormones while suffer severe pain; and (iv) willing to sign the informed consent. Patients were randomized into the meridian-sinew (MS) group (63 patients) and anatomy group (61 patients), and all patients were identified by Identification Scale of TCM Constitutions [17]. The following criteria were for exclusion: (i) those who received hormone therapy in the past 3 months; (ii) those who received intraarticular hyaluronic acid injection in the past 6 months; (iii) those who received joint irrigation or joint endoscopy in the past year; (iv) those with diseases hindering the safe participation in the experimental design and affecting the completion of the study, for example, they suffered from myocardial infarction or stroke, congestive heart failure, severe chronic obstructive pulmonary disease, cancer, diabetes, and other serious systemic diseases as well as serious mental illness in the past 3 months; (v) those who had a medical history or clinical manifestation of hemorrhagic tendency, including the use of anticoagulants at that time; (vi) those with inflammatory arthritis (such as rheumatoid or psoriatic arthritis); (vii) Those who were also involved in other research; (viii) Those who underwent a knee replacement surgery; (ix) those who used to participate in the treatment related to research on knee osteoarthritis in the past; (x) those who were excessively fear of acupotomy therapy; (xi) those who were receiving local external therapy at the same time, such as local application therapy; (xii) those who cannot complete various scales; (xiii) those who were unwilling to participate in random grouping. Patients who met any of the above items were excluded [20].

**Figure 1:**
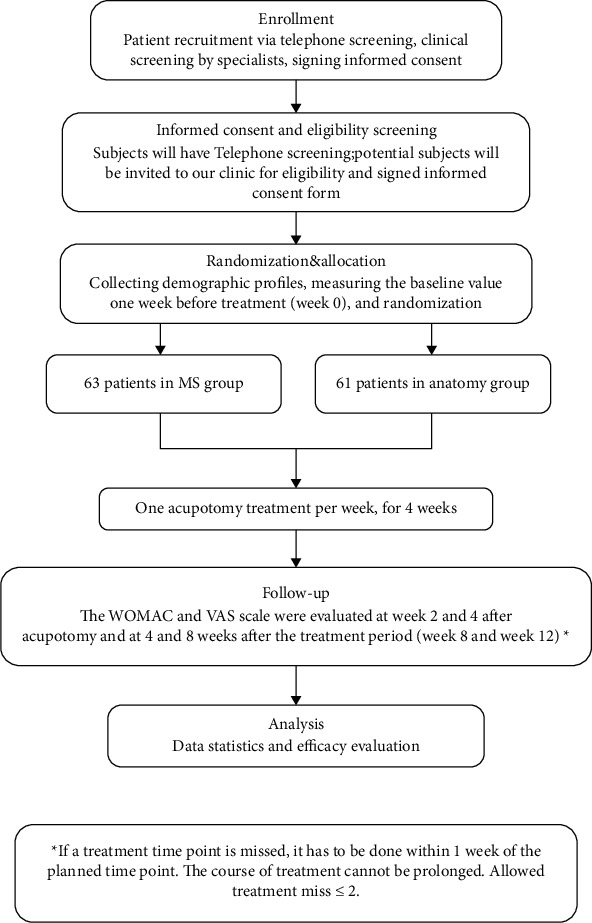
Flow diagram of the recruitment process, group allocation, and participation in the two interventions. All participants who completed a follow-up were included in the corresponding analysis.

Patients were randomly assigned to the meridian-sinew group or anatomy group through computer-generated random numbers. The eligibility of KOA subjects will be assessed by clinical physicians. The intervention will be implemented by experienced acupuncturists. The subjects and assessors will be blinded to subjects' allocations. The acupuncturist will do not take part in assessments or data entry. Data will be analyzed by an independent statistician (Figure 1).

### 2.2. Study Design Flowchart

Figure 1 shows the recruitment process, group allocation, and participation in the two interventions.

### 2.3. Interventions

#### 2.3.1. Patients in the MS Group

**Figure 2:**
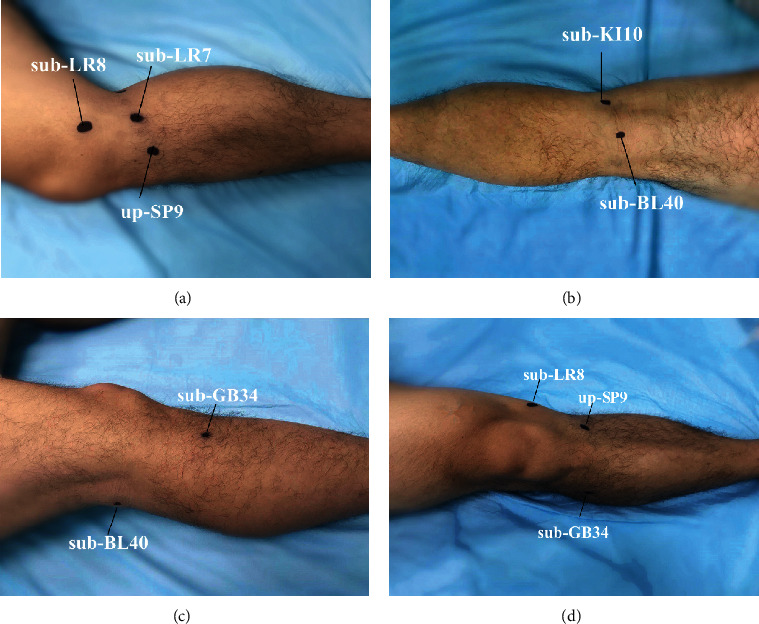
Acupoints in the MS group.

**Figure 3:**
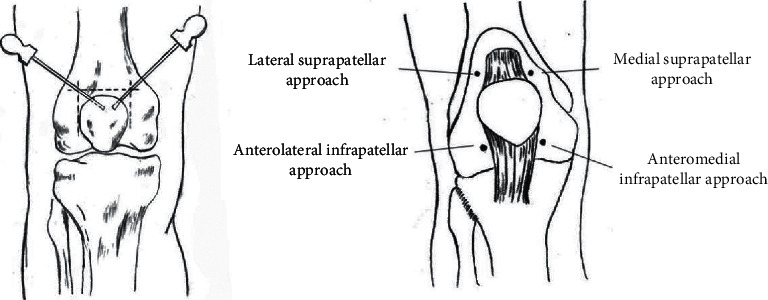
Schematic diagram of acupoint selection through tendons.

**Figure 4:**
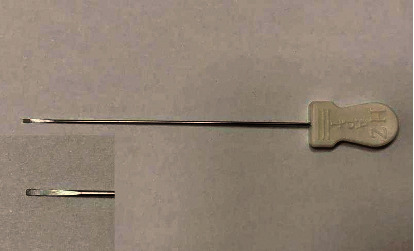
The needle knife used in acupotomy.

Body position: the patients were asked as supine position for the anterior, medial, and lateral release, with the knee joint flexion of about 30° and a soft pillow under the knee; the prone position was adopted for the posterior release, with a soft pillow in front of the ankle. Point locating: selected tender point and cord-like node along the three Yang meridians of the foot and three Yin meridians of the foot as positive reaction points, select sub-BL40, sub-KI10, sub-GB34, sub-LR8, sub-LR7, and up-SP9 (standard tendons disease lesions) if tender point and cord-like node were absent, and mark the selected points with a marker (see Figures 2 and 3). Disinfection and anesthesia: operators used iodophor to disinfect the treatment area and 0.5% lidocaine (1 ml) for local infiltration anesthesia on each point. Operation: the operator inserted the disposable needle knife (0.8 mm × 40 mm No. 4, Hanzhang, Figure 4) until it reached the surface of the bone and released the adhesions mainly with vertical movements and then with subcutaneous weeping motion. Local pressing hemostasis was applied right after removal of needle knife, and then bandages in the same position. This was performed once a week for 4 weeks. Assessment on week 2 and week 4 were done after acupotomy.

#### 2.3.2. Anatomy Group

Body position: the patients were asked as the same positions as the MS group. Point locating: attachments of medial and lateral collateral ligament, attachments of patellar ligament, tendon insertion of quadriceps femoris, and anserine bursa (8 points in total) were selected according to Foundations of Acupotomy and Clinical Acupotomy [21]. Disinfection and anesthesia: the operator used iodophor to disinfect the treatment area; and 0.5% lidocaine (1 ml) for local infiltration anesthesia on each point. Operation: needle knives were inserted perpendicularly and then follow the four-step procedure of acupotomy—location, orientation, pressing-releasing, and puncture. Local pressing hemostasis was applied right after removal of the needle knife and then bandages in the same position.

This was performed once a week for 4 weeks. Assessment on week 2 and week 4 was done after acupotomy.

The causes of acupotomy-related adverse reactions, including hemorrhage, subcutaneous hemorrhage, hematoma, syncope, severe pain, and local infection, were recorded during the study and follow-up period.

### 2.4. Outcomes and Measures

#### 2.4.1. Outcomes

The primary outcome was WOMAC score change from baseline. The secondary outcomes were VAS change from baseline.

#### 2.4.2. Measurement Instruments

The WOMAC (Likert version, a 5-point rating scale with a score range of 0–4; worst score: 4) was measured at baseline (week 0, one week before treatment), week 2, week 4, week 8, and week 12.

VAS (a 11-point rating scale with a score range of 0–10; worst score: 10) was used to measure pain intensity, and measurement was taken on week 0, week 2, week 4, week 8, and week 12.

Identification Scale of TCM Constitutions (a scale consisted of approximately 60 choice questions) questionnaire was used to identify each subject's TMC constitutions accompanied with Chinese medicine specialists' diagnosis. It was expected to recognize if there is any potential relationship between clinic outcome and TCM constitution.

### 2.5. Data Analysis

Estimation of sample size was based on the results of previous studies and then calculated according to the calculation formula for sample size estimation. The formula is as follows: noninferiority test: *n* = [ × (*U*_*α*_ + *U*_*β*_) × *P* (1 − *P*)]/*δ*^2^ = *C*1 × *P* (1 − *P*)/*δ*^2^ [22], where *δ* is the equivalent standard (boundary value), *U*_*α*_ and *U*_*β*_ are the one-sided standard normal deviation bound value, *n* is the sample size, *P* is the average effective rate, and *C*1 and *C*2 are the coefficient terms. Take *α* = 0.05, and *β* = 0.1 (test efficiency power = 1–0.1 = 0.90). The sample size is estimated to be 104 cases. According to the clinical research purpose, practical operability, and 20% sample loss, the final number of observation samples of the research group is 124 cases.

Data were analyzed by SPSS 22.0. Comparisons of continuous variables between the MS and anatomy groups will be assessed using the independent sample *t*-test. Comparisons of categorical data between the two groups were tested by the *χ*^2^ test. Within group variances of different time points were analyzed by one-way ANOVA. *P* < 0.05 was used as the standard for statistical significance.

## 3. Results

### 3.1. Subjects

One hundred twenty-four patients aged from 40 to 74 were enrolled in this study, including 41 men (33.1%) and 83 women (66.9%). 44 patients' TCM constitution were identified as Yin-Yang harmony (35.5%), 43 as Yang deficiency (34.7%), and 37 as others (39.8%). Demographic information and TCM constitutions of each group are given in Table 1, and several patients' data were absent in the “knee joint degeneration” and “age at first onset” sets.

### 3.2. Primary and Secondary Outcomes

**Figure 5:**
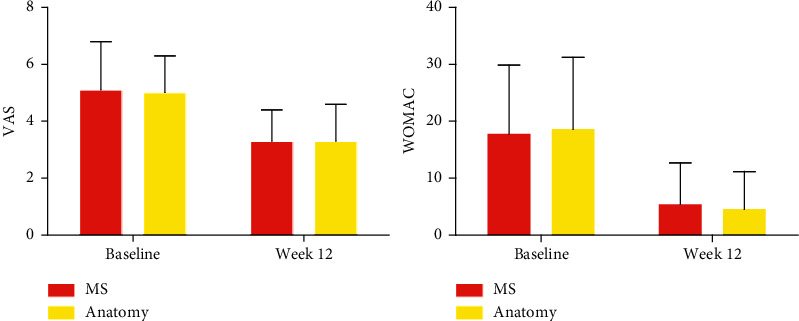
VAS and WOMAC scores at baseline and week 12 of the MS and anatomy groups.

VAS (*F* = 22.61, *p* < 0.01) and WOMAC (*F* = 24.84, *p* < 0.01) scores declined with time in both the MS and anatomy groups. Compared with the anatomy group, there was no statistically significant difference in VAS or WOMAC scores at baseline or during treatment (*p* > 0.05) (Table 2 and Figure 5).

**Figure 6:**
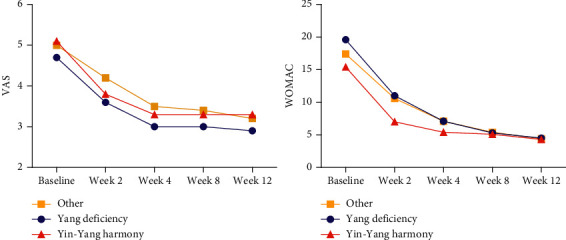
Repeated measures of VAS and WOMAC in subgroups of different constitutions.

Similarly, no significant differences were observed in VAS and WOMAC between different constitution subgroups of each group (Table 3 and Figure 6).

At the end of the treatment, VAS decreased by 1.7 and 1.8 in the MS group and anatomy group, respectively, and there was no statistically significant difference (*t* = 0.28, *p*=0.78). WOMAC decreased by 12.3 and 13.0 in the MS group and anatomy group, and no significant differences between the two groups (*t* = 0.28, *p*=0.78) (Table 4).

### 3.3. Adverse Reactions

Five patients reported adverse reactions related to acupotomy, including 5 cases of subcutaneous hemorrhage at the acupotomy site and 1 case of tingling after acupotomy. All adverse reactions were reported as mild and did not require special medical intervention. All patients recovered completely from the adverse reactions and completed the trial.

## 4. Discussion

KOA is one of the most common diseases among the elderly. Acupotomy as a traditional Chinese treatment showed excellent clinical effectiveness in the treatment of KOA [23]. Specialist could use a “meridian-sinew scope” to make the treatment easier by observing the inner structure of the knee joint directly. Though such scope may not be available in some sites, the cost could be expensive. In primary hospitals, specialists usually conduct acupotomy on the basis of the anatomy theory (without a scope) or traditional meridian-sinew theory. The meridian-sinew theory was first seen in the meridian-sinew chapter of the Yellow Emperor's Internal Classic (Huangdi Neijing). Previous studies have shown that this theory has been applied to relieve knee joint pain in the treatment of KOA [14, 24]. One study' [14] treatment time was too short, while the total duration of the study was 12 weeks and the duration of treatment was 4 weeks. The sample size was also too small to assess overall changes in the disease state, while the meridian-sinew release group, acupuncture group, and control group are, respectively, 27 cases, 26 cases, and 26 cases. Another study [24] compared the efficacy of sinew acupuncture with sham acupuncture. In their study, they used needle noninsertion as the control, which may produce a smaller, nonspecific effect compared to needle-insertion sham controls. This trial will expand their knowledge of whether sinew acupuncture will reduce pain intensity, improve the symptoms and movements of KOA patients, and improve QOL. Our previous study [18] is to study safety and effectiveness of the treatment of knee osteoarthritis with acupotomy therapy. We found both acupotomy therapies guided by the meridian sinew theory and by the anatomy theory of Western medicine have a good curative effect on knee osteoarthritis, but acupotomy guided by the meridian-sinew theory has more superiorities in operability, safety, and effectiveness, which is easy to be generalized in grass-roots and community hospitals. Our study combined acupotomy with the meridian-sinew theory and added Identification Scale of TCM Constitutions.

This study was conducted to explore the effectiveness of acupotomy based on the meridian-sinew theory in the treatment of KOA compared with acupotomy based on anatomy theory. There are 6 meridian sinews around the knee, and the reactive sites (tender point and cord-like node along the three Yang meridians of foot and three Yin meridians of foot, or sub-BL40, sub-KI10, sub-GB34, sub-LR8, sub-LR7, and up-SP9 if tender point and cord-like node were absent) located along the course of the 6 meridian sinews were chosen to be the treatment sites in this study.

This study showed that the VAS and WOMAC score improved in both groups, and there were no significant differences between MS and anatomy groups nor between subgroups of TCM constitutions.

In the health management of KOA patients, TCM constitution identification and TCM intervention are introduced. Under the guidance of the theory of “prevention of disease,” personalized TCM health management schemes are formulated for different TCM constitution types, and health management modes suitable for China's national conditions and with TCM characteristics are discussed to achieve the purpose of active prevention and reduce the risk of KOA. It can consolidate the therapeutic effect. This study revealed that Yin-Yang harmony and Yang deficiency were prone to knee osteoarthritis, and the curative effect after acupuncture was better. Several cases of postacupuncture pain were also relieved quickly after rest, while the pain of Yin deficiency patients increased after needle knife treatment, and the rest time was about 2 hours. However, because there was only one patient with Yin deficiency, it could not be concluded that Yin deficiency patients could not use needle knife.

However, this study has several limitations. First, results of a small sample size and a short treatment and follow-up period cannot represent a long-term efficacy or adverse effect profile. Second, the WOMAC and VAS estimated in the study both represent subjective perceptions of the patients, though indicators such as interleukin, tumor necrosis factor, and matrix metalloproteinases levels could reflect objective responses. Third, the results of subscales (pain, stiffness, and physical function) of WOMAC between two interventional groups have not been reported, and we will report in our future research.

## 5. Conclusion

According to the WOMAC and VAS results of this study, acupotomy based on the meridian-sinew theory has an equivalent effectiveness to acupotomy based on anatomy theory on pain relief, joint function improvement in spite of patients' TCM constitution, and has no adverse effects in the treatment of KOA. In summary, acupotomy based on the meridian-sinew theory is a reliable, safe, convenient, and low-cost treatment in KOA treatment.

## Figures and Tables

**Table 1:** Demographic information and TCM constitutions of the MS and anatomy groups.

Group		Total number	MS (patients (%))	Anatomy (patients (%))	*χ* ^2^	*P*
Age (year)	≤ 60	41	25 (44.6)	31 (55.4)	1.722	0.189
	>60	83	26 (57.8)	19 (42.2)		

Gender	Man	41	23 (56.1)	18 (43.9)	0.686	0.407
	Woman	83	40 (48.2)	43 (51.8)		

Constitutional type	Yin-Yang harmony	44	23 (52.3)	21 (47.7)	0.511	0.774
	Yang deficiency	43	20 (46.5)	23 (53.5)		
	Others	37	20 (54.1)	17 (45.9)		

Knee joint degeneration	Left knee	31	17 (54.8)	14 (45.2)	0.024	0.988
	Right knee	41	22 (53.7)	19 (46.3)		
	Both knees	19	10 (52.6)	9 (47.4)		

Age at first onset	≤ 60	68	35 (51.5)	33 (48.5)	0.535	0.465
	>60	25	15 (60.0)	10 (40.0)		

^*∗*^MS, meridian-sinew; TCM, traditional Chinese medicine.

**Table 2:** Repeated measures of VAS and WOMAC in different treatment groups ($\bar{x}\pm s$).

Index	Group	Baseline	Week 2	Week 4	Week 8	Week 12	*F*_time_ (*p*)	*F*_group_ (*p*)	*F*_time∗group_ (*p*)
VAS	MS	5.1 ± 1.7	4.1 ± 1.2	3.7 ± 1.1	3.5 ± 1.0	3.3 ± 1.1	22.61 (<0.01)	0.61 (0.43)	1.35 (0.25)
	Anatomy	5.0 ± 1.3	3.9 ± 1.7	3.3 ± 1.6	3.3 ± 1.2	3.3 ± 1.3			

WOMAC	MS	17.8 ± 12.1	10.6 ± 8.9	7.8 ± 7.8	6.6 ± 7.7	5.4 ± 7.3	24.84 (<0.01)	0.40 (0.81)	0.06 (0.81)
	Anatomy	18.6 ± 12.6	10.8 ± 11.6	7.1 ± 9.3	5.3 ± 7.0	4.6 ± 6.6			

^*∗*^MS, meridian-sinew; TCM, traditional Chinese medicine; VAS, visual analog scale/score; VAS pain score, 0–10; lower score = better outcome. WOMAC pain score, 0–20. It was assessed with the following five items: pain during walking, stair climbing, resting, weight bearing, and pain at night. Each subscale used the following descriptors: none (0 points), mild (1 point), moderate (2 points), severe (3 points), and extreme (4 points).

**Table 3:** Repeated measures of VAS and WOMAC in patients with different constitution of traditional Chinese medicine ($\bar{x}\pm s$).

Subgroup		Baseline	Week 2	Week 4	Week 8	Week 12	*F*_time_ (*p*)	*F*_group_ (*p*)	*F*_time∗group_ (*p*)
VAS	Yin-Yang harmony	5.1 ± 1.5	3.8 ± 1.0	3.3 ± 1.1	3.3 ± 1.1	3.3 ± 1.1	31.27 (<0.01)	1.86 (0.16)	0.83 (0.58)
	Yang deficiency	4.7 ± 1.5	3.6 ± 1.6	3.0 ± 1.5	3.0 ± 1.1	2.9 ± 1.2			
	Others	5.0 ± 1.5	4.2 ± 1.9	3.5 ± 1.6	3.4 ± 1.3	3.2 ± 1.3			

WOMAC	Yin-Yang harmony	15.4 ± 11.5	7.0 ± 5.5	5.4 ± 5.5	5.1 ± 5.0	4.3 ± 4.7	28.22 (<0.01)	0.94 (0.40)	0.78 (0.62)
	Yang deficiency	19.6 ± 13.9	11.0 ± 11.9	7.1 ± 10.2	5.3 ± 8.7	4.5 ± 7.9			
	Others	17.4 ± 12.6	10.6 ± 10.8	7.1 ± 8.2	5.4 ± 7.1	4.4 ± 6.9			

VAS, visual analog scale/score; VAS pain score, 0–10; lower score = better outcome. WOMAC pain score, 0–20. It was assessed with the following five items: pain during walking, stair climbing, resting, weight bearing, and pain at night. Each subscale used the following descriptors: none (0 points), mild (1 point), moderate (2 points), severe (3 points), and extreme (4 points).

**Table 4:** Comparison of VAS and WOMAC decline values ($\bar{x}\pm s$).

Subgroup		VAS				WOMAC			
		*n*	Decrease	*t/F*	*P*	*n*	Decrease	*t/F*	*P*
Group	MS	63	1.7 ± 1.6	0.28	0.78	63	12.3 ± 13.7	0.29	0.77
	Anatomy	60	1.8 ± 1.8			59	13.0 ± 12.7		

Subgroup	Yin-Yang harmony	44	1.8 ± 1.7	0.15	0.85	44	10.8 ± 11.9	0.95	0.38
	Yang deficiency	42	1.8 ± 1.6			43	14.8 ± 14.9		
	Others	36	1.6 ± 1.8			36	12.4 ± 12.5		

VAS, visual analog scale/score; VAS pain score, 0–10; lower score = better outcome. WOMAC pain score, 0–20. It was assessed with the following five items: pain during walking, stair climbing, resting, weight bearing, and pain at night. Each subscale used the following descriptors: none (0 points), mild (1 point), moderate (2 points), severe (3 points), and extreme (4 points).

## Data Availability

The datasets generated during the current study are available from the corresponding author upon request.
